# The Biomechanics of Cervical Spondylosis

**DOI:** 10.1155/2012/493605

**Published:** 2012-02-01

**Authors:** Lisa A. Ferrara

**Affiliations:** OrthoKinetic Testing Technologies, LLC, Southport, NC 28461, USA

## Abstract

Aging is the major risk factor that contributes to the onset of cervical spondylosis. Several acute and chronic symptoms can occur that start with neck pain and may progress into cervical radiculopathy. Eventually, the degenerative cascade causes desiccation of the intervertebral disc resulting in height loss along the ventral margin of the cervical spine. This causes ventral angulation and eventual loss of lordosis, with compression of the neural and vascular structures. The altered posture of the cervical spine will progress into kyphosis and continue if the load balance and lordosis is not restored. The content of this paper will address the physiological and biomechanical pathways leading to cervical spondylosis and the biomechanical principles related to the surgical correction and treatment of kyphotic progression.

## 1. Introduction

Cervical spondylosis is a common progressive degenerative disorder of the human spine often caused by the natural aging process. It is defined as “vertebral osteophytosis secondary to degenerative disc disease” due to the osteophytic formations that occur with progressive spinal segment degeneration [1–3]. Early spondylosis is associated with degenerative changes within the intervertebral disc where desiccation of the disc occurs, thus causing overall disc height loss and a reduction in the ability of the disc to maintain or bear additional axial loads along the cervical spine [3, 4]. At birth, the intervertebral discs are healthy, with the proteoglycan matrix within the nucleus pulposus maintaining a 70% to 90% water content which declines with aging [4]. As the water content declines within the nucleus pulposus, the once healthy glistening gelatinous appearance changes into a darkened and discolored fibrous “crabmeat” consistency with a loss in water content and a loss in the structural integrity.

**Figure 1:**
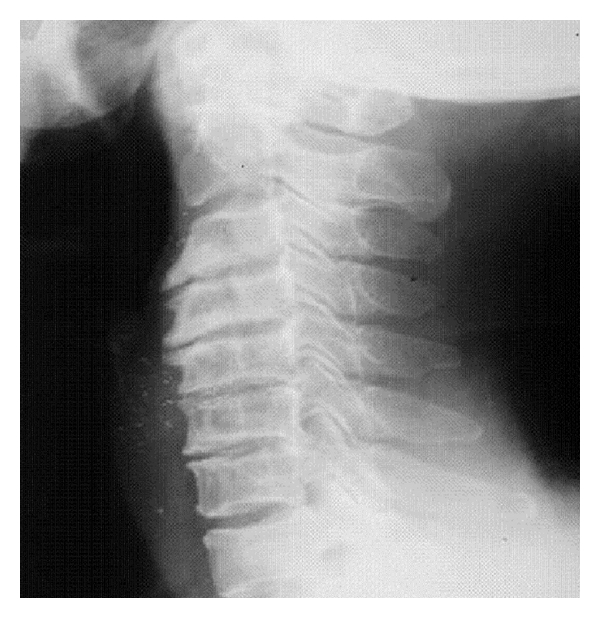
Radiographic representation of cervical spondylosis.

Once the disc starts to degenerate and a loss in disc height occurs, the soft tissue (ligamentous and disc) becomes lax, resulting in ventral and/or dorsal margin disc bulge and buckling of the ligaments surrounding the spinal segment, accompanied by a reduction in the structural and mechanical integrity of the supportive soft tissues across a cervical segment. As the ventral column becomes compromised, there is greater transfer of the axial loads to the uncovertebral joints and also along the dorsal column, resulting in greater loads borne by the facet joints. As axial loads are redistributed to a greater extent along the dorsal column of the cervical spine, the facet joints are excessively loaded resulting in hypertrophic facets with possible long-term ossification of the posterior longitudinal ligament [1]. When the load balance of the cervical spine is altered and disrupted, as is the situation with cervical degeneration, the remaining functional and supportive structures along the cervical spinal column will absorb the added stress that is transferred to the surrounding structures and adjacent levels along the spine. Eventually these structures will also be excessively loaded, resulting in a cascade of events of further degeneration and tissue adaptation. Overloading the soft tissues and bone eventually causes osteophytes to form in response to excessive loading in order to compensate for greater stresses to the surrounding bone and soft tissue (Figure 1).

Cervical spondylosis presents itself in three symptomatic forms as neck pain, cervical radiculopathy, and cervical myelopathy. Neck pain and cervical radiculopathy (nerve root involvement) can be acute, subacute, or chronic conditions resulting from various stages along the degenerative cascade [1]. Cervical myelopathy is less frequent in the spondylotic patient and occurs in older patients with symptoms such as neck, subscapular, or shoulder pain, accompanied by shock sensations and numbness in the extremities [1, 5–7]. Cervical myelopathy involves motor and reflex changes indicative of a more chronic condition and can eventually result in spastic weakness and numbness of the extremities, loss of dexterity, spastic gait, dorsal column function loss, and painful paresthesias [1, 6, 8, 9]. These chronic symptoms can eventually become permanent with poor prognosis. 

## 2. Pathogenesis and Etiology of Cervical Spondylosis

The primary cause of cervical spondylosis is age-related degeneration. However, there are some exceptions where spinal injuries to the disc can augment the degenerative process in the younger patient. A secondary manifestation of spondylosis is related to the compression of the vascular and neural structures caused by a loss in the disc height and impinging osteophytes that contribute to the numbness, shock-like sensations, pain, and chronic motor and sensory affects, which if not corrected may lead to permanent disabilities.

**Figure 2:**
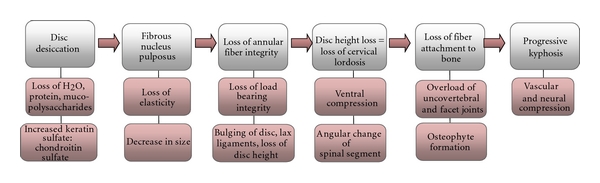
Pathophysiological and biomechanical pathway of cervical spondylosis.

It is this physiological degenerative cascade that contributes to the biomechanical changes that can cause neural and vascular compression, pain, and loss of function.  Figure 2 illustrates the chain of events of cervical spondylosis starting with the biomechanical changes that can result in neural and vascular compression. Early changes in the proteoglycan matrix cause an increase in the ratio of keratin sulfate to chondroitin sulfate resulting in the loss of water within the disc. This desiccation causes the nucleus pulposus to lose elasticity, shrink in size, and lose the ability to bear axial loads. Since the dorsal fibers of the annulus are thinner than the ventral aspect, there is a path of least resistance through the annulus for a nucleus pulposus herniation. The annular fibers become mechanically compromised with further disc desiccation and are unable to effectively maintain axial loads, causing buckling of the spinal ligaments and annular fibers under compressive loads, which are further exacerbated with eccentric loads (i.e., flexion, torsion, and bending) [1–3]. The resultant loss in disc height causes the discs to bulge, the ligamentous tissue to become lax and buckle, and the ventral aspect of the cervical spine to compress. At this point, there are significant alterations in the load distribution along the cervical spinal column, with an end result of kyphosis of the cervical spine. If not reversed, the kyphosis will continue to progress, the annular and Sharpey's fibers will separate from the vertebral periphery and bony endplates, resulting in reactive bone formation where the fibers have been separated. These resultant bone spurs can be formed along the ventral or dorsal margin of the cervical spine and within the canals in response to the altered biomechanical loads, causing compression of the neural and vascular structures.

The unique properties of bone and soft tissue are the ability to regenerate and remodel the tissue along the lines of loading and stress application, thus regaining the structural integrity. However, if the load balance along the spinal column is altered and is not restored, the tissue will remodel along the altered load and stress planes, causing the tissue to remodel along new planes of loading. Since osteophyte or bone spur formation will occur in response to excessive eccentric loads, new bone will form in areas of greater stress and will be resorbed in areas of less stress.

The loss in the axial load bearing capabilities of the degenerative segment leads to a disruption in the load transfer along the neutral axis of the spinal column, also known as a change in the overall load balance, thereby transferring greater loads to the uncovertebral and facet joints, further accelerating the formation of spurs and osteophytes into the surrounding foramen, with greater angulation of the cervical spinal column ventrally. The ventral angulation along the cervical spine is a continuous cascade of mechanical events. As the lordotic angle is reduced, the moment arm about the center of rotation or instantaneous axis of rotation (IAR) is increased, therefore, changing the overall sagittal angulation and reducing the spinal canal diameter [1, 3, 4, 9].

## 3. Histologic and Immunohistochemical Findings with the Spondylotic Disc

Elegant studies have been performed to characterize the histological and immunohistochemical differences between cervical disc herniation and spondylosis. Disc herniation can be an early contributor to spondylosis, as herniation creates a loss in the mechanical integrity of the intervertebral disc due to the extrusion or bulging of the nucleus pulposus through compromised annular fibers. The herniation often occurs dorsally, as the dorsal annular fibers are thinner and provide a less resistant pathway for the compromised nucleus pulposus matter. The intervertebral discs with surrounding tissues, subchondral vertebral bone, cartilaginous endplate, and posterior longitudinal ligaments were collected en bloc during decompression surgeries in 198 patients presenting with cervical intervertebral disc herniation resulting in 248 discs for evaluation. An additional 252 discs were harvested in a similar manner from 166 patients presenting with cervical spondylosis to provide a histological and immunohistochemical assessment between cervical spondylosis and disc herniation [10]. The disc-herniated patients were younger (49.9 years, range 25–78 years) than the cervical spondylosis (mean age of 59.6 years, ranging from 32 to 83 years), with all patients presenting with signs and symptoms of radiculopathy, myelopathy, or myeloradiculopathy with an average duration of symptoms prior to surgery of 3.2 months. The control discs (free from cervical radiculopathy and myelopathy) were harvested during autopsies taken from eight donors with a mean age of 73 years. Chondrocyte proliferation, a change in the granular matrix, fibrocartilage degeneration of the annulus fibrosus and nucleus pulposus, cell proliferation, cartilage disorganization, cracks, microfractures, sclerotic endplates, and vascularization of the disc were parameters used to grade the level of disc degeneration. The herniated cervical discs demonstrated granulation tissue with new vascularization and an infiltration of CD68-positive macrophages surrounding the herniated tissue with greater advanced degeneration in the outer layer of the annulus [10]. The spondylotic discs demonstrated thicker bony endplates and tumor necrosis factor and matrix metalloproteinase with greater advanced degeneration in the cartilaginous endplates and inner layer of the annulus. In essence, there were distinct differences and markers for distinguishing herniated discs from spondylotic discs.

## 4. Biomechanics of the Spondylotic Spine

**Figure 3:**
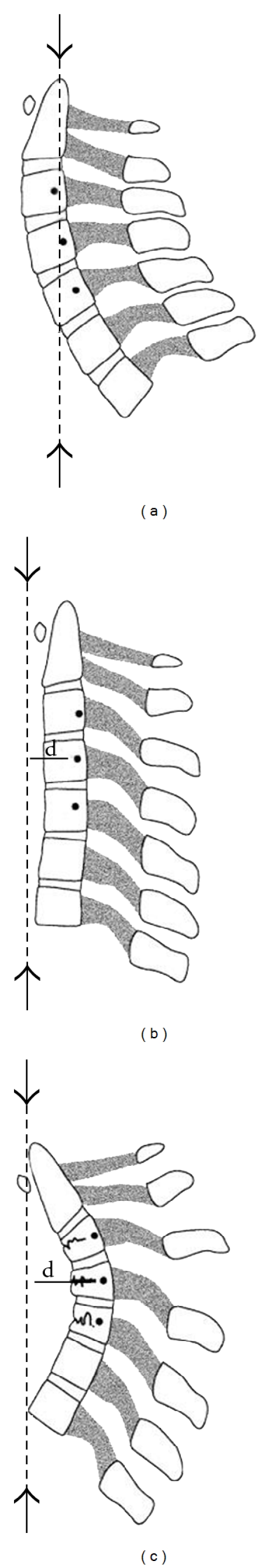
Cervical spine postural changes related to the degenerative process will lead to the spondylotic spine. In the normal lordotic posture of the cervical spine, the axial force is applied along the instantaneous axis of rotation (IAR) with no deviation from this neutral point of rotation (a). However, with early disc height loss, the lordotic posture is reduced and the axial force is now offset from the instantaneous axis of rotation (d) causing a moment arm at this point of rotation. If an axial force is placed at a particular distance from the center of rotation or the IAR, a bending moment is applied about this point (b), and it will take less force to induce injury to the apical spinal segment. The larger a moment arm, the greater the bending moment, which will cause further progression of the kyphosis (c). (Taken from Benzel EC: Biomechanics of Spine Stabilization. Rolling Meadows, American Association of Neurological Surgeons Publications, 2001 [6].)

Kyphotic deformity with neural and vascular compression often accompanies cervical spondylotic myelopathy. Initially the loss of disc height as a consequence of disc desiccation and altered load transmission along the cervical spine can lead to postural changes (Figure 3(b)). As spinal cycling continues during activities of daily living, the disc will continue to lose height ventrally. This altered posture will result in an increased moment arm about the point of central rotation or the IAR. In the healthy spine, axial loads are applied along the IAR and the loads are supported along the ventral column of the spine. However, with altered posture, the axial load profile along the cervical spine changes as the ventral column (vertebral bodies, disc, and ligamentous tissue) can no longer maintain these loads, and there is a transfer of the loads and stresses to the surrounding bony elements. Loss of the lordotic posture induces a greater moment arm at the point of rotation (point d in Figures 3(b) and 3(c)) when an axial load is applied. Without restoration of the native lordotic posture which will restore the “load balance” along the cervical spine, further axial loading will induce further progression of the kyphotic posture (Figure 3(c)).

Halting the progression of kyphosis related to cervical spondylosis is the main objective for surgical intervention and can be treated through ventral surgical fixation or through a combined ventral and dorsal approach of stabilizing fixation. Ventral approaches for surgical correction of this disorder provide the necessary ventral column support to resist further compression and angulation of the cervical spine and can provide improved decompression of the neural and vascular structures. 

**Figure 4:**
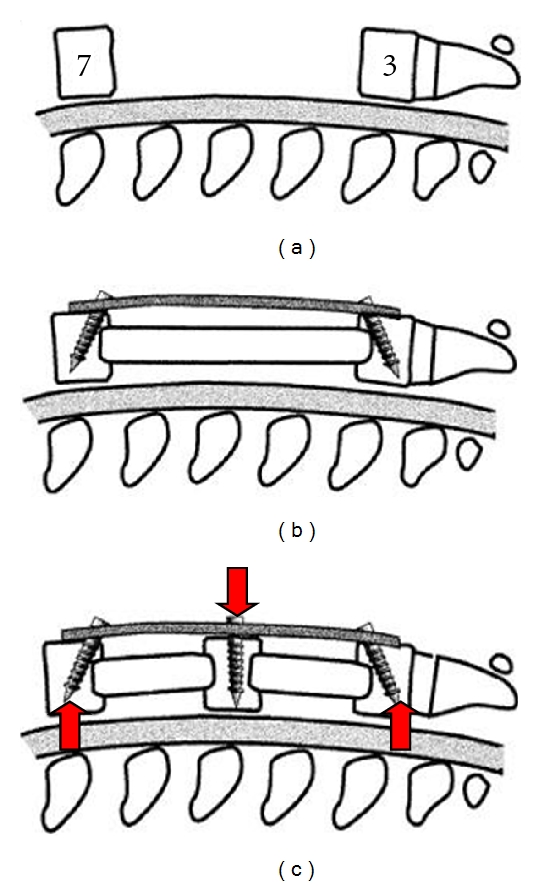
Ventral corpectomy approach with one large strut grafts and two points of screw fixation for kyphotic deformity correction. A large strut graft without multiple points of fixation may not maintain the lordotic posture and can subside into the vertebral endplates, resulting in loss of fixation and loss of the lordotic restoration. However, multiple interbody fusions with supplemental plate fixation and multiple points of screw fixation distribute the loads over greater points of fixation, thereby reducing the risk to localized stress risers, providing improved resistance to translation, loss of lordosis, and subsidence. (Taken from Benzel EC: Biomechanics of Spine Stabilization. Rolling Meadows, American Association of Neurological Surgeons Publications, 2001 [6].)

Numerous in vitro and in vivo studies have confirmed that approximately 80% of the axial load is transmitted along the ventral column of the human spine with improved resistance to higher axial loads and better biomechanical stability when ventral column stabilization is employed [1, 3, 4, 11]. A biomechanically challenging kyphotic posture may require 360° of correction which provides both ventral and dorsal column support to the degenerative sites. However, the disadvantages with ventral fixation occur when suboptimal bone quality is present, as is often the case with these patients. For both the ventral and dorsal surgical approaches used for kyphotic deformity correction, poor bone quality contributes to suboptimal screw purchase into the surrounding bone, compromising the long-term stability. Furthermore, a long strut graft across a corpectomy for ventral fixation provides a long lever arm with one strut of bone that does not conform to the native lordotic posture of the patient's cervical spine and has an increased risk to subsidence and dislodgment out of the site, resulting in a loss of the lordotic correction. Therefore, multisegmental ventral fixation, such as consecutive intervertebral fusion grafts supplemented with ventral plate fixation, provides multiple points of fixation for better load distribution across each fixation point along the cervical spine. Multiple interbody fusions with multiple screw fixation of the plate will provide better restoration and long-term stability of the lordotic posture, while also providing neural decompression (Figure 4). Mechanically, this configuration provides a three-point bend application of opposing forces to the compromised spinal segment(s) to provide improved maintenance of the lordotic posture and greater resistance to translational loads (arrows in Figure 4). Posterior fixation can also accompany segmental ventral fusions, provided the posterior fixation does not offload the interbody fusion grafts. Posterior fixation combined with ventral support can provide similar translational resistance and three-point bend stability for kyphotic correction.

## 5. Summary

Fusion of the degenerative unstable spine is often a final alternative to alleviate a painful spinal segment. Ventral interbody fusion is incorporated in a fusion construct to maximize the axial load bearing capacity of the spine while limiting the pathologic motion across a spinal segment. In situations such as kyphotic deformity correction, multisegmental ventral interbody fusions can provide the necessary ventral column support incorporating multiple points of fixation to resist the demanding translational, rotational, and bending loads placed upon on the degenerative kyphotic cervical spine and will provide ample strength to maintain the restored lordotic posture. Ventral support using multiple interbody fusion grafts supplemented with dorsal fixation across each level will also provide similar mechanical attributes as that of ventral support, where both approaches towards kyphotic deformity will allow for better force distribution across many points of fixation, thus minimizing the risk of stress risers that can cause graft subsidence, expulsion, screw loosening, or loss of fixation. Biomechanically, these strategies will improve the restoration of the lordotic posture for long-term fixation and stability. By restoring the lordotic posture of the cervical spine, the load balance is restored, where 80% of the axial loads transmitted along the ventral column is aligned at the IAR of the spinal column, effectively, halting the progression of the kyphotic curvature. Segmental fixation results in reduced localized forces and stresses at each spinal level and across the instrumentation, with reduced stresses on each screw and across each graft site for improved long-term fixation.
